# Differences and similarities in breast and colorectal screening participation: a spatial and temporal analysis to reveal intervention areas, France

**DOI:** 10.1016/j.pmedr.2026.103518

**Published:** 2026-06-02

**Authors:** Cindy M. Padilla, Anais Mazurier, Loïc Kwekeu, Nirmala Prajapati, Patricia Soler Michel, Maud Ottavy

**Affiliations:** a UMR U1309/RSMS, EHESP (French School of Public Health), Rennes, France; b CRCDC, Regional Cancer Screening Center, Lyon, France; c Environment and Lifestyle Epidemiology Branch, International Agency for Research on Cancer, World Health Organization, Lyon, France

**Keywords:** Breast cancer screening, Colorectal cancer screening, Mapping_space-time pattern, Determinants, Intervention

## Abstract

**Objective:**

Although breast and colorectal cancers are included in organized screening programs, participation remains suboptimal and influencing factors have evolved. We present a reproducible method for visualizing the spatiotemporal patterns of participation in these screening programs.

**Methods:**

The study includes breast and colorectal screening participation rates for women aged 50–74 years across 751 census blocks residing in the Rhône and Lyon metropolitan areas. Over two periods (pre, 2015–2019 and post, 2020–2022 pandemic), this ecological study allowed the creation of strategic maps to identify local priority areas for interventions. A spatial statistic Local Moran's I was used to identify clusters of high and low participation in both periods. These results were combined to map temporal evolution, classifying census blocks as consistently high, low, improved, or worsened in participation. Contextual factors have helped to describe these trends.

**Results:**

Maps revealed clear spatial disparities. For colorectal screening, participation increased by about 5 percentage points, from 33% to 38%, while breast screening remained stable at around 49%. In Lyon metropole, low-income census blocks with good healthcare access exhibited persistently low participation in both screenings. In the Rhône area, participation improved over time, and healthcare providers availability appeared to be a key determinant.

**Conclusions:**

This approach visualizes screening participation across space and time, providing a tool for policymakers to identify areas requiring intervention and adapt prevention strategies to local contexts.

## Highlights

• Map is a reproducible method for visualizing spatiotemporal dynamics of screenings.
• Reveals differences and similarities in the spatial disparities of both screenings.
• Economic vulnerability is a key barrier, especially in urban areas.
• New perspectives are needed to target intervention areas and prevention strategies.

## 1 Introduction

Breast cancer and colorectal cancers are major contributors to global cancer-related mortality, with an estimated 626,679 and 881,000 deaths, respectively, worldwide in 2018 (International Agency for Research on Cancer, 2020). In France in 2018, breast cancer was the most frequently diagnosed cancer (58,459 new cases), ahead of colorectal cancers (20,120 cases), and ranked first among all cancer deaths in women (14,434 deaths), followed by lung and colorectal cancers (7908 deaths).

Given their high incidence and severity, the prevention of breast and colorectal cancers should be a public health priority. Screening is an excellent preventive intervention for the early detection of cancer and can improve patient prognosis for both breast and colorectal cancers (Broeders et al., 2012). Women who get screened are 40% less likely to be diagnosed with late-stage breast cancer (Giordano et al., 2012). In Europe, population-based mammography screening has been associated with a 25%–31% reduction in mortality, and a 38%–48% reduction among women receiving adequate follow-up (Lauby-Secretan et al., 2018; Lauby-Secretan et al., 2015). Similarly, more than 95% of colorectal cancer cases could benefit from surgical treatment if diagnosed at an early stage of the premalignant polyp, leading to a significant reduction in mortality (Altobelli et al., 2014).

Participation in both screening programs in France remains suboptimal. In 2021–2022, 34.2% of the target population underwent colorectal cancer screening, and 44.8% received a screening mammogram. A decreasing trend in mammography use has been observed since 2010, reaching 48.5% in 2019, across all age groups and regions, whereas the colorectal cancer screening rate has remained stable. These low participation rates are particularly concerning given recent trends in cancer incidence: an upward trend in breast cancer incidence was observed between 2010 and 2023 (61,214 cases, +0.3% variation), ahead of colorectal cancer incidence in women (21,370 cases, +0.4%). Since 2004, women aged 50–74 has been invited by letter to have a mammogram free of charge every two years. Since 2008, men and women aged 50–74 have been invited every two years to perform a stool-based test (fecal immunochemical test (FIT) from 2015), followed, if positive, by a colonoscopy.

Previous studies conducted in Belgium and Switzerland have highlighted regional variations in participation in cancer screening programs, demonstrating both interrelationships between programs and geographical disparities in participation (Ferrari et al., 2022). In France, most studies have focused on single screening program, and few have simultaneously examined breast and colorectal screenings or their temporal evolution (Données globales d'épidémiologie des cancers, 2025).

To address this gap, this study describes differences and similarities in participation in mammography and colorectal cancer screening from 2015 to 2022 in the Lyon metropolitan area and Rhône department, examining spatial patterns, temporal consistency, and shared contextual determinants at the census block level.

## 2 Methods

### 2.1 Study design and population

A descriptive ecological study design was used to assess geographical variation in participation in mammography and colorectal cancer screening (CRCS) within the Auvergne-Rhône-Alpes (AURA) region. The study focuses on the Rhône department combining two contiguous local authorities: the predominantly urban Lyon metropolitan area (Lyon MA) and the mostly rural Rhône area (Rhône dpt), comprising 1,843,300 inhabitants (Département du Rhône: deux collectivités, un million d'emplois − Conseil départemental du Rhône et Métropole de Lyon: deux collectivités à compétences départementales | Insee, 2025). These study areas can be divided into 751 IRIS, including 511 in the Lyon metropolitan area and 240 in the Rhône area. The IRIS is the smallest geographical and administrative unit for which socio-economic and demographic data are available in France. Defined by the French National Institute for Statistics and Economic Studies (INSEE) (INSEE: National Institute of Statistics and Economic Studies, n.d), it corresponds to a French sub-municipal census block of approximately 2000 inhabitants.

Census blocks without any eligible women (for example, an industrial census block or a park) and census blocks with less than 10 eligible women were excluded. The final dataset included 730 census blocks (492 for the Lyon MA and 238 for the Rhône dpt).

Screening participation data (between 2015 and 2022) come from the Regional Cancer Screening Coordination Centre (CRCDC) of the AURA region (Depistage des cancers - Auvergne Rhône Alpes [Internet], 2022) and were analyzed conducted at the aggregated (census block) level. For mammography, participation rates were calculated by dividing the number of participating women by the number of eligible women aged 50–74 variable by couple of year (in 2021–2022, *N* = 243.856 women invited through the national screening program to undergo free mammography and *N* = 129.457 women participated). For colorectal cancer, participation rates were calculated by dividing the number of women aged 50–74 who underwent screening using the fecal immunochemical test (FIT) by the number of eligible women invited (in 2021–2022, *N* = 193.214 women invited and *N* = 72.906 women participated). The study focused exclusively on women to ensure a homogeneous target population and to allow comparison of participation patterns in mammography and colorectal cancer screening.

In March 2020, the World Health Organization declared a global pandemic of Covid_19. In response, cancer screening programs were paused in many jurisdictions worldwide as part of broader suspensions of non-emergency healthcare services (Mayo et al., 2021; Lofters et al., 2023; Wang et al., 2026). In France, although monitoring of organized screening programs continued, invitations to eligible women were suspended due to the temporary closure of screening facilities. Afterwards, a business continuity plan was implemented, along with raising people's awareness of their health. For this study, two distinct periods were defined: a pre- Covid_19 period, comprising the years 2015–2016 to 2018–2019, and a post- Covid_19 period, comprising the years 2020 to 2022. Temporal patterns participation in both screening programs from 2015 to 2022 in the Lyon MA and the Rhône dpt, are presented in the supplementary graph.

### 2.2 Measures

**Fig. 1:**
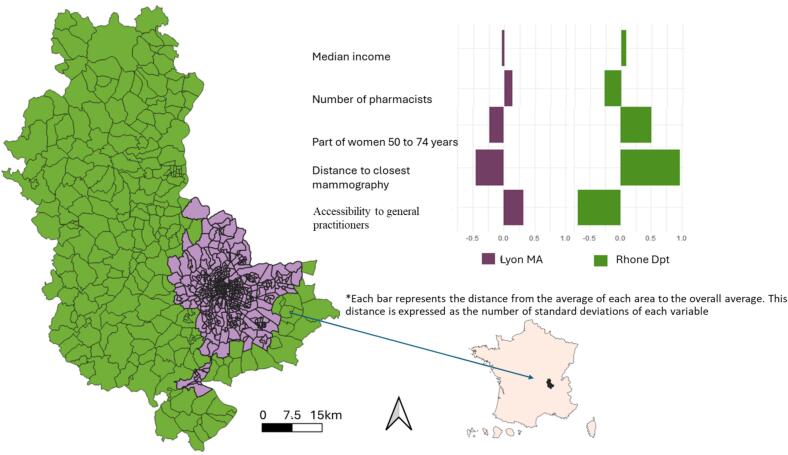
In the Lyon metropolitan (purple) and the Rhône department (green) areas, characteristics description of each in comparison to the global study area average according to their sociodemographic and healthcare accessibility level in 2020 using the standardized profile graph. (For interpretation of the references to colour in this figure legend, the reader is referred to the web version of this article.)

Screening participation is influenced by a variety of factors. Individuals living in the same neighborhood often share similar lifestyles, which may influence their participation in screening programs (Vallée et al., 2010). Previous studies have shown that poor geographic accessibility to health services, limited availability of general practitioners and high levels of deprivation, are key factors influencing healthcare utilization (Duport et al., 2008; Soffian et al., 2021). Improved characterization of these factors in relation to participation patterns may help to better understand screening behaviors. Geographic accessibility was estimated by calculating travel time (in minutes) from the centroid of each geographical unit to the nearest mammography service. To describe the availability of general practitioners**,** localized potential accessibility was obtained at the municipality level. This indicator reflects the density of health professionals in full-time equivalents, standardized by sex and age distribution of the population. It based on the enhanced two-step floating catchment area method (Mangeney and Lucas-Gabrielli, 2019). A sensitivity analysis was conducted, and median income alone was retained as the most relevant proxy for the socioeconomic dimension. Data at the census block level were obtained from the 2020 national census. Fig. 1 presents territorial profiles for the Lyon MA (in purple) and the Rhône dpt (in green).

### 2.3 Statistical analysis

The proposed steps present a simple and easily reproducible method for visualizing target areas for intervention to improve screening participation. The first step consisted in testing the spatial autocorrelation of screening participation rates using Moran's I index. When spatial autocorrelation is present, clusters of census blocks with high or low participation rates may be identified (Li et al., 2007). The global Moran's I statistic for spatial autocorrelation strength is represented as follows:

Moran'sI=nΣiΣjWijyi−y¯yj−y¯ΣiΣjWijΣiyi−y¯2=nΣiΣjWijyi−y¯yj−y¯S2ΣiΣjWij

where yi and yj represent participation rates in census blocks i and census blocks j, n is the total number of census blocks y is the mean of observations, S2 is the variance of the observation variable, and Wij is the element in the spatial weights matrix. The Moran's value lies in the range of [− 1 (negative spatial autocorrelation),1 (positive autocorrelation)]. In our case, we defined contiguity using the ‘queen’ option, called ‘contiguity edges corners’. Geographically contiguous census blocks may exhibit similar screening participation patterns due to spatial diffusion of health behaviors through informal communication and observational learning. These mechanisms may influence health-seeking behavior (Chuang et al., 2013) and, through spillover effects, could contribute to the geographical clustering of mammography screening participation. Previous studies (Prajapati et al., 2022) have highlighted the importance of such spillover effect in explaining variation in cancer screening utilization rates.

Then, a local autocorrelation step is carried out to identify clusters of high and low participation in two periods before and after the Covid_19 (Anselin, 1995). This process enables us to determine for each period, low-to-low cluster, high-to-high clusters and high-to-low, low-to-high clusters. A low-to-low cluster of census blocks indicated census blocks with a below-average participation rate that were also surrounded by census blocks of below-average adherence rates. To do this, an autocorrelation indicator, LISA (Local Indicator of Spatial Autocorrelation), was developed. The result is the z-score and the associated statistical *p*-value. The LISA statistic is represented as follows, where yi, yj, Wij and S2 are the same as those used for Moran's I index.

$$\mathrm{LISA}=\frac{\mathrm{y\mathrm{i}}-\bar{y}}{S^{2}}\sum w_{\mathrm{ij}}\left(\mathrm{yj}-\bar{y}\right)$$

We then combined both periods to map temporal changes by classifying census blocks into four categories: worsened trend, corresponding to areas with initially insignificant or high participation that shifted to low participation; consecutive low, referring to areas with persistently low participation across both periods; consecutive high, indicating consistently high participation; and improved trend, defined as areas with initially insignificant or low participation that shifted to high participation in the second period.

All analyses were conducted using R software v4.4.1.

## 3 Results

Overall participation is higher for mammography (ranging from 51.8% in 2015–2016 to 53.1% in 2021–2022) than for CRCS (ranging from 31.5% in 2015–2016 to 37.7% in 2021–2022). In the Lyon MA, the average mammography participation rate remained stable (pre-Covid_19: 49.4 ± 6.9%, and post-Covid_19:49.8 ± 8.4%), whereas CRCS participation increased from 30.5% ± 5.4% to 35.4% ± 7.8%. For the Rhône dpt, average participation increased for both screenings between the two periods: from 55.5% to 59.5% (+4 percentage points) for mammography, and from 37.2% to 42.5% (+5.3 percentage points) for CRCS.

### 3.1 Geographic distribution and clusters of both screenings

**Fig. 2:**
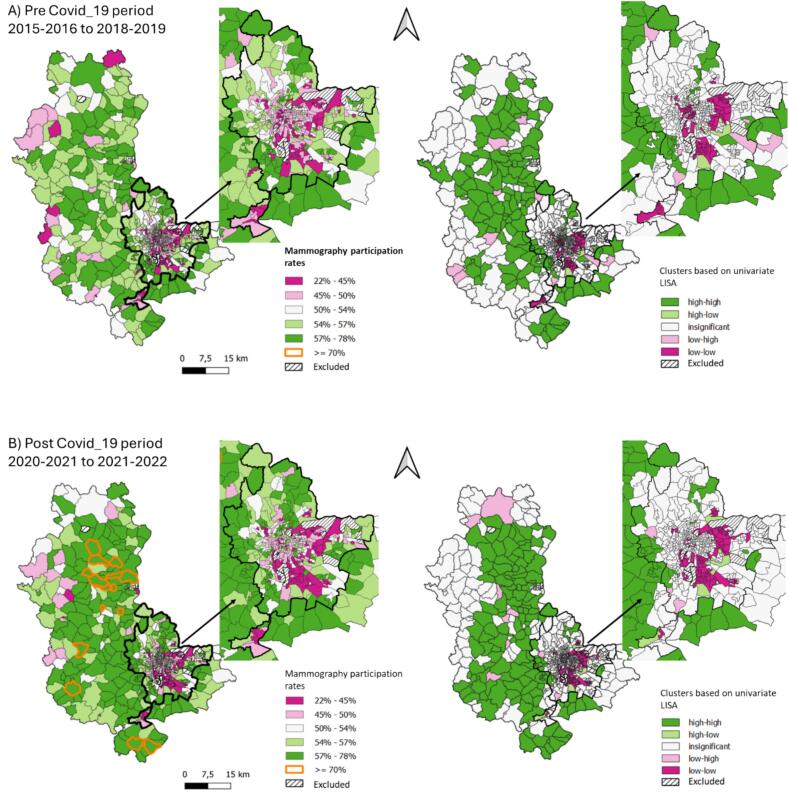
In the Lyon metropolitan and the Rhône dpt areas during A) pre and B) post Covid_19 periods. **Left**: Maps of mammography screening participation by census block (pink to green shading indicates lower to higher uptake). **Right**: Clusters of high participation (green: high–high denote areas with significantly above-average uptake) and clusters of low participation (pink: low–low denote areas with significantly below-average uptake). (For interpretation of the references to colour in this figure legend, the reader is referred to the web version of this article.)

**Fig. 3:**
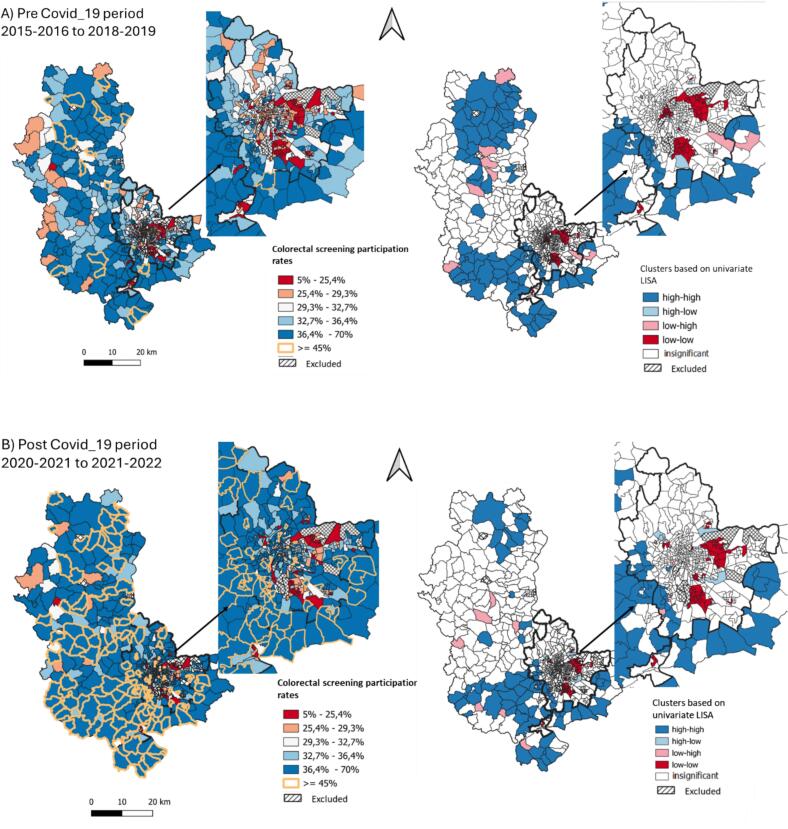
In the Lyon metropolitan and the Rhône dpt areas during A) pre and B) post Covid_19 periods. **Left:** Maps of colorectal screening participation by census block (Red to blue shading indicates lower to higher uptake). **Right:** Clusters of high participation (blue: high–high denote areas with significantly above-average uptake) and clusters of low participation (red: low–low denote areas with significantly below-average uptake). (For interpretation of the references to colour in this figure legend, the reader is referred to the web version of this article.)

The global Moran's values for mammography (range 0.41–0.55) and colorectal cancer screening (range 0.42–0.48) were all positive and significant, indicating the presence of spatial clusters in both screenings. Fig. 2, Fig. 3 present the geographic distribution of mammography and CRCS participation rates, and their clusters of participation detected pre and post Covid_19. LISA maps revealed similar clusters of census blocks with low participation rates for both mammography and CRCS in Lyon MA, while clusters with high participation rates for both screenings were in the southern part of the Rhône dpt. This pattern is also reflected in the participation maps, showing an increase in darker areas, and areas exceeding the European regulatory threshold in the second period.

### 3.2 Mapping of the spatiotemporal screening participation patterns

**Fig. 4:**
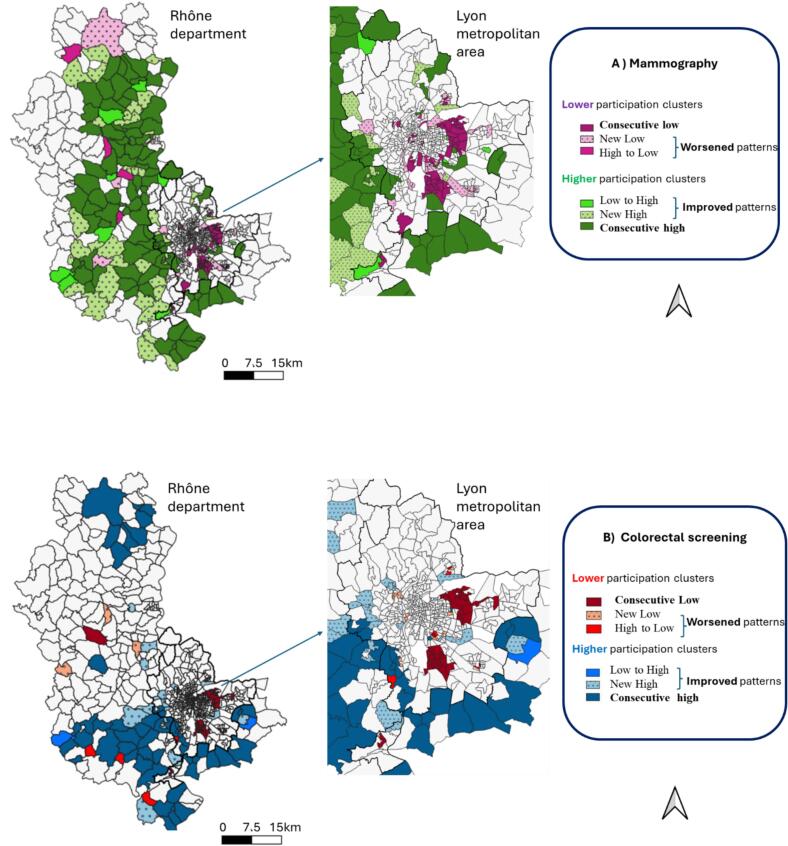
Strategic spatiotemporal mammography A) and colorectal B) screenings participation maps of the Lyon metropolitan and the Rhône dpt areas between pre and post Covid_19 periods (Worsened trend, corresponding to areas that shifted to low participation; consecutive low, referring to areas with persistently low participation across both periods; consecutive high, indicating consistently high participation; and improved trend, defined as areas that shifted to high participation in the second period).

Fig. 4 presents in one map the temporal trends of clusters of screening participation, using the LISA statistics, between the two time periods. The average participation rates according to the spatiotemporal patterns (worsened, consecutively low, consecutively high or improved) are provided in the supplementary table. The maps highlight clusters of census blocks with similar temporal trends characterized by consistently high (census blocks with statistically higher participation than the average in both periods) or low participation (census blocks with statistically lower participation than the average in both periods) in mammography and CRCS programs. In Lyon MA, dark pink (14.2%) and red (10.5%) census blocks, present persistently low participation rates in mammography and CRCS despite multiple interventions. These blocks are in the eastern suburbs (municipalities of Vaulx-en-Velin, Vénissieux and Villeurbanne). In the southern part of the Rhône dpt, some census blocks, located in Saint-Symphorien-d'Ozon and the Mornant district, persistently showed high participation rates in both screenings throughout the study period, represented by dark green and blue areas.

The distribution of census blocks and their breast and colorectal average screening participation rates according to their spatiotemporal screenings participation patterns were explored in Lyon MA and the Rhône dpt (supplementary table). In Lyon MA, 8.3% of census blocks (located in Lyon city 8th arrondissement, St Priest and Rilleux la Pape) exhibited a worsened participation trend for mammography compared to only 3.4% for CRCS (*p* = 0.013). In contrast, in the Rhône dpt sustained high participation is more pronounced for mammography (35.7% of census blocks) than for CRCS (17.6% of census blocks) (*p* = 0.004). The improved participation patterns were predominantly observed for mammography (15.5%) compared to CRCS (4.2%) (*p* = 0.027) between the two periods.

Socioeconomic status appears to play a more significant role than geographic proximity in predicting screening behavior within the urban setting of the Lyon MA (Table 1). In Lyon MA, census blocks with consecutively low participation in both screenings have low average median income levels (Mammography: €17,078; CRCS: €16,816), despite relatively high general practitioners' availability and short distances to mammography services. Conversely, census blocks with consecutively high or improving participation exhibit higher income levels (up to €28,772). In the Rhône dpt, healthcare supply seems a more relevant determinant of screening participation. Census blocks with high or improved mammography and CRCS participation between the two periods showed higher general practitioners' availability and shorter distances to mammography services.

**Table 1:** Average of the contextual census blocks measures grouped by breast and colorectal screening participation trends between pre and post Covid_19 periods, in the Lyon metropolitan and the Rhône department areas.

	Lyon MA (*N* = 492)					Rhone Dpt (*N* = 238)				
	Breast screening (mean ± SD)			Colorectal screening (mean ± SD)		Breast screening (mean ± SD)			Colorectal screening (mean ± SD)	
	Availability of general practitioners	Distance to mammography	Median income	Availability of general practitioners	Median income	Availability of general practitioners	Distance to mammography	Median income	Availability of general practitioners	Median income
Worsened trend	4.51 ± 1.38	1.79 ± 0.95	19,446 ± 4326	4.20 ± 0.67	19,713 ± 4259	2.65 ± 1.13	17.24 ± 14.07	23,022 ± 2293	2.49 ± 1.13	25,780 ± 2661
Consecutively low	4.54 ± 1.38	1.66 ± 0.82	17,078 ± 3022	4.68 ± 1.63	16,816 ± 2377	–	–	–	–	–
Consecutively high	3.67 ± 0.63	2.61 ± 2.32	28,372 ± 3359	3.61 ± 0.82	27,903 ± 3316	3.30 ± 1.03	10.81 ± 7.08	25,691 ± 2755	3.04 ± 1.13	25,403 ± 2741
Improved trend	4.16 ± 0.77	2.99 ± 1.87	26,075 ± 5230	3.93 ± 1.01	28,079 ± 4500	2.95 ± 1.01	14.71 ± 8.41	24,652 ± 2554	3.17 ± 1.05	27,361 ± 3223

## 4 Discussion

This study provides new insights into the spatial and temporal dynamics of participation in mammography and colorectal cancer screening across the Rhône department and the Lyon Metropolitan Area between 2015 and 2022. Whilst overall participation rates were consistently higher for breast cancer screening than for colorectal cancer screening, these results highlighted marked geographical disparities and diverse findings in terms of participation levels and trends over time. Furthermore, the temporal analysis provides further insight into trends in participation, enabling interventions to be stepped up where participation is low or is declining.

This study yielded noteworthy findings, with consistently low participation in both screening programs over time in the peri-urban communities of Lyon MA (e.g., Vaulx-en-Velin, Vénissieux, Villeurbanne). Despite multiple interventions implemented in these areas, participation rates have not yet reached the desired results. The results showed that census blocks with low screening rates were also characterized by the lower average median income. The Lyon MA is a center for economic decision-making, with a high proportion of jobs in the tertiary sector, high unemployment and blue-collar workers, low income and education levels, and a high concentration of immigrants. These findings are consistent with previous studies, which reported that a higher percentage of foreigners (Ferrari et al., 2022) or greater concentration of specific ethnic groups (Buehler et al., 2019) is associated with a lower probability of participating in colorectal and mammography screenings. These findings suggest that perceived language barriers and discomfort with discussing screening are key factors, potentially leading to difficulties in understanding health information and translating it into appropriate preventive behaviors (Durand et al., 2021). Moreover, population living in deprived areas —characterized in our study by low average median income —often face a greater burden of complex health conditions, which may result in preventive measures, including cancer screening, being deprioritized in daily health management. In France, Prajapati et al., reported that screening participation fell with increasing level of deprivation (Prajapati et al., 2022), and similar findings were found in England (Lal et al., 2021), Canada (Lofters et al., 2023) and other settings (de Klerk et al., 2018; Kurani et al., 2020; Jessiman-Perreault et al., 2023).

In Lyon city center and the southern suburbs (Saint-Fons), the maps also point out census blocks where mammography participation remains low. Opportunistic screening was not considered in this study, but a previous study (Padilla et al., 2019) conducted by our team in 2015–2016 showed that a higher proportion of opportunistic screening took place in the city center and in Saint-Fons, and that the clusters of low participation observed in these areas may reflect this phenomenon. In such cases, participation rates in organized screening programs may be underestimated.

In contrast, results highlight distinct patterns of screening participation in the predominantly rural Rhône dpt areas. Clusters of high participation for both screenings were observed in the southern part, whereas significant improvement over time were noted only for mammography participation in the northern part. This finding may indicate a need for enhanced health promotion regarding the benefits and procedures of colorectal screening, particularly targeting individuals approaching or in their 50s. We hypothesized that the promotional actions implemented since 2010 on this territory have been effective, thanks to the strong commitment of local authorities (the municipality and the community of municipalities) to a multiyear, multisectoral approach (CDC, 2025). The population in Rhône dpt is well integrated into the labour market with a low unemployment rate and less pronounced inequalities in living standards.

The divergent temporal trends between mammography and colorectal cancer screening also merit attention. Our results show that screening participation changed following the pandemic, with some census blocks exhibiting worsened trends and others showing improvements. Observed declines in participation may reflect healthcare system disruptions, including temporary suspension of screening programs, reduced capacity, and reallocation of healthcare resources during the pandemic (Wang et al., 2026). Reductions in participation may also be explained by care avoidance behaviors, driven by fear of infection and reduced willingness to attend healthcare facilities (Wenger et al., 2022). Additionally, preventive services such as screening were often deprioritized in favor of acute care, contributing to temporary declines in participation during the pandemic. Post-pandemic recovery trajectories varied across regions, potentially contributing to heterogeneous spatiotemporal patterns of participation.

Our study illustrates how the distribution of healthcare accessibility and supply differs between the urban Lyon MA and the predominantly rural Rhône dpt. In the Rhône dpt**,** census blocks with unfavorable participation trends had, on average, greater distances to mammography services and lower general practitioners' availability than those with favorable trends, suggesting that the level of healthcare supply may influence screening participation. Availability and continuity of care may play a pivotal role, resulting in a seamless and sustained patient–provider relationship. Previous studies have shown up, mediated through patient–provider communication, consistent delivery of screening reminders, and increased trust in medical advice influences adherence to colorectal cancer screening. In urban Lyon MA, economic vulnerability may have a greater impact on screening participation than geographic accessibility. In France and Quebec, an inverse urban-rural relationship has been previously described (Prajapati et al., 2022). In 2022, a systematic review investigated the combined effects of geographic access and socioeconomic characteristics reported that, in urban contexts, geographic accessibility has less influence participation on mammography participation than socioeconomic level (Conti et al., 2022).

Although this study provides finding on spatiotemporal patterns of mammography and colorectal screening participation, important limitations should be considered. First, only women eligible for the organized screening program were included in the calculations of participation rates at the census block level, and opportunistic screening was not considered. Additionally, this study did not account for health facilities located near women's workplaces as potential sites for screening. Although we have identified changes in participation trends following the Covid-19 period, the lack of individual-level data on these women's perceptions and experiences prevents us from fully understanding these behaviors. Further analytical studies and individual-level research could help better assess the role of socioeconomic conditions and access to healthcare in these decisions.

Approaches that identify suboptimal screening participation across territories using hotspot detection are often to a single screening program (Soffian et al., 2021), and comparative analyses between mammography and colorectal screening remain sparser in the literature (Ferrari et al., 2022; Jessiman-Perreault et al., 2023). Our study addresses this gap by presenting a temporal approach dimension combined with the spatial approach to better understand shifting participation patterns and to inform screening promotion strategies. Although significant research has focused on the geographic identification of screening participation clusters (Feng et al., 2016; Bermudez et al., 2022; Kim et al., 2023; Duncan et al., 2016), the temporal component of these underlying screening participation distributions has been largely ignored.

Our descriptive study is the first stage of a project aimed at gaining understanding of screening participation in this département in France. In future research, we plan to complement these findings with an individual-level qualitative approach, incorporating the perspectives of residents from the territories highlighted in this analysis. Residents' perception and individual characteristics are important determinants of their decision to participate in screening.

## 5 Conclusion

Mapping has long been a fundamental tool in public health. By visualizing risk factors across a study area, health geography analyses can help predict local future screening trends and guide prevention strategies. These analyses are easily reproducible across different times points and locations, providing a framework to evaluate and compare screening participation. Moreover, integrating contextual characteristics of the census blocks with mapping findings can generate hypotheses for further analytical or individual-level research. Such information can support policymakers in designing targeted interventions for areas with lower cancer screening participation rates.

## CRediT authorship contribution statement

**Cindy M. Padilla:** Writing – original draft, Supervision, Investigation, Funding acquisition, Conceptualization. **Anais Mazurier:** Methodology, Formal analysis, Data curation. **Loïc Kwekeu:** Methodology, Formal analysis, Data curation. **Nirmala Prajapati:** Writing – review & editing, Validation. **Patricia Soler Michel:** Writing – review & editing, Visualization, Validation, Data curation, Conceptualization. **Maud Ottavy:** Writing – review & editing, Validation, Supervision, Methodology, Data curation, Conceptualization.

## Funding

This work was supported by the French National Cancer Institute (INCa) [grant numbers INCA_18620, 2023]. The funders did not contribute to the writing of the article nor to the collection or analysis of the data.

## Declaration of competing interest

The authors declare the following financial interests/personal relationships which may be considered as potential competing interests: The authors declare that they have no competing interests. Where authors are affiliated with the International Agency for Research on Cancer/World Health Organization, the authors alone are responsible for the views expressed in this article and they do not necessarily represent the decisions, policy, or views of the International Agency for Research on Cancer/World Health Organization.

## Data Availability

The data that has been used is confidential.
