# Supplementary material for: Differences and similarities in breast and colorectal screening participation: a spatial and temporal analysis to reveal intervention areas, France
Source: Prev Med Rep. 2026 Jun 2;67:103518. doi: 10.1016/j.pmedr.2026.103518 (PMC13267630; doi:10.1016/j.pmedr.2026.103518)
Supplement: Supplementary material [file mmc1.docx]

**Supplementary table 1**: Repartition of census blocks and their average participation rates grouped by breast and colorectal screening participation patterns between pre and post Covid_19 periods, in the Lyon Metropolitan and the Rhône department areas.

| **Spatiotemporal participation of**  **census blocks** | **Lyon MA (N=492)** | | | |  | **Rhone DPT (N=238)** | | | |
| --- | --- | --- | --- | --- | --- | --- | --- | --- | --- |
|  | **Breast screening** | | **Colorectal screening** | | | **Breast screening** | | **Colorectal screening** | |
|  | Number of CB (%) | Average participation rates  in 2015 & 2021 | Number of CB (%) | Average participation rates  in 2015-2021 | | Number of CB (%) | Average participation rates in 2015-2021 | Number of CB (%) | Average participation rates in 2015-2021 |
| Worsened trend | 41 (8.3%)* | 48.3%-43.3% | 17(3.4%)* | 30.2% - 27.8% | | 8(3.6%) | 53.7%- 51.3% | 6(2.5%) | 33.5%-28.3% |
| Consecutively low | 70(14.2%) | 42.4%- 37.9% | 52(10.5%) | 23.2% - 25.2% | | 0% | - | 0% |  |
| Consecutively high | 14(2.8%) | 57.1% - 59.9% | 11(2.2%) | 37.8%- 48.2% | | 85(35.7%)* | 57.5%- 63.2% | 42(17.6%)* | 40.5% - 47.3% |
| Improved trend | 11(2.2%) | 51.7% - 56.8% | 10(2.0%) | 34.2%-42.7% | | 37(15.5%)* | 54.3%- 61.4% | 10(4.2%)* | 34.8%- 51% |

The number of census blocks according to their spatiotemporal cancer screenings participation patterns are presented in %. The average screening participation in 2015 and 2021 are presented in %.

* Significant *p*-values of the comparison between breast and colorectal cancer screenings were obtained from chi-squared or Fisher's exact test according to their theory frequencies. Participation data come from the Regional Cancer Screening Coordination Centre (CRCDC) of the AURA region aggregated at the census block level.

**Supplementary figure 1**. The screening participation rates from 2015 to 2022 in the Lyon metropolitan and the Rhone department areas, France.
